# Viro-immunological characterization of naïve patients with high cerebrospinal fluid (CSF) HIV RNA

**DOI:** 10.7448/IAS.17.4.19710

**Published:** 2014-11-02

**Authors:** Francesca Iannuzzi, Francesca Bai, Esther Merlini, Mattia Trunfio, Lidia Borghi, Teresa Bini, Antonella d'Arminio Monforte, Giulia Marchetti Carla

**Affiliations:** Clinical of Infectious Disease, San Paolo Hospital, University of Milan, Milan, Italy

## Abstract

**Background:**

HIV can spread into the central nervous system (CNS) early in the course of infection and this turns into intrathecal inflammation and neuronal damage. We aimed to investigate clinical and immunological parameters associated with elevated CSF VL in HIV-infected ART-naïve patients.

**Materials and Methods:**

HIV+ ART-naïve patients underwent a comprehensive battery of neurocognitive (NC) tests and lumbar puncture (LP) for CSF HIV-RNA detection. Plasma HIV-RNA and peripheral T-cell immune-phenotypes (CD38/CD45RA/CD45R0/CD127 on CD4/CD8) were also assessed (flow cytometry). High-CSF HIV RNA was defined as≥10000cp/mL (H-CSF), while CSF HIV RNA<10000cp/mL characterized low VL patients (L-CSF). Chi-square and Mann-Whitney tests were used. Parameters independently associated with CSF VL were explored by multivariate regression.

**Results:**

A total of 131 patients were retrospectively enrolled. Forty-two patients (32%) had CSF VL >10000 cp/mL.

Table 1 shows the features of H- versus L-CSF patients. Compared to L-CSF patients, H-CSF patients displayed lower current CD4+%, lower CD4/CD8 ratio and higher CD8%. No differences in NC tests performance were observed between groups (p=0.6). Regarding T-cell immuno-phenotypes, H-CSF patients displayed a higher proportion of CD45R0+CD38+CD8+ (11 vs 7%, p=0.02) and lower expression of CD45RA+CD8+ % (16 vs 20%, p=0.007), in comparison to L-CSF patients. In multivariate analysis CD45RA+CD8+ T-cells % (OR 0.917, CI 95% 0.852–0.987, p=0.002) was associated with H-CSF, even after adjustment for plasma VL, CD8 and CD4 count. Globally, in univariate CSF VL inversely correlated with CD45RA+CD8+ % (r=−0.223, p=0.0217) and CD127+CD4+ % (r= −0.204, p= 0.0225), while a positive association was found between CSF and plasma VL (r=0.303, p=0.0004) and CD8 % (r=0.211, p=0.016). In multivariate linear regression, in addition to positive association between plasma and CSF VL (β: 0.212, 95% CI 0.02–0.41, p=0.032), also CD45RA+CD8+ % were confirmed inversely associated to CSF VL (β: 0.21, 95% CI −0.5 to −0.002, p=0.036), adjusting for CD4/CD8 and CD4CD127 %.

**Conclusions:**

We hereby describe a 32% prevalence of H-CSF in a cohort of HIV+ ART-naïve patients. Subjects with high-CSF viral replication are mostly with higher systemic immune activation, in particular the percentage of naïve CD8 T-cell is positively associated with CSF VL, irrespective of plasma VL. In HIV+ ART-naïve patients, especially if featuring a hyperactivated T-cell immune-phenotype, lumbar puncture should be considered to further guide CNS-targeted cART.

**Table 1:** Demographic and immune-virological characteristics of the study population and comparison between H-CSF and L-CSF patients

	Tot naïve n=131	CSF-HIV-RNA <10000 cp/mL (L-CSF pts) (n=89)	CSF-HIV_RNA >=10000 cp/mL (H-CSF pts) (n=42)	P value
Female, n (%)*	12 (9)	6 (6)	6 (16)	0.162
Age (years), median (IQR)*	38 (32–45)	38 (32–45)	38 (32–49)	0.886
Time since first HIV diagnosis (months), median (IQR)*	3.7 (1–21)	4.4 (1.3–15.9)	3 (1–33)	0.718
Plasma HIV-RNA, Log cp/mL median (IQR)*	4.89 (4.22–5.42)	4.69 (4.16–5.26)	5.23 (4.78–5.85)	0.002
CSF-HIV-RNA, Log cp/mL median (IQR)*	3.65 (3.04–4.19)	3.44 (2.89–3.67)	4.76 (4.22–5.09)	0.0001
CD4+ T-cell, cell/mmc median (IQR)	307 (150–417)	320 (154–446)	267 (125–366)	0.076
CD4+ T-cell, % median (IQR)	19 (11–24)	20 (12–25)	17 (10–20)	0.028
CD8 T-cell, cell/mmc, median (IQR)	921 (650–1172)	901 (650–1092)	1037 (652–1222)	0.207
CD8 T-cell, % median (IQR)	57 (51–66)	55 (49–62)	62 (53–73)	0.005
Nadir CD4 t-cell, cell/mmc median (IQR)*	282 (130–388)	305 (131–405)	209 (125–357)	0.157
Ratio CD4/CD8, median (IQR)*	0.33 (0.17–0.45)	0.37 (0.2–0.48)	0.28 (0.14–0.37)	0.021
Symptomatic for headache, n (%)	14 (11)	4 (5)	10 (24)	0.001
HCV co-infection, n (%)*	7/112 (6)	5/76 (6)	2/36 (5)	0.834
HBC co-infection, n (%)*	8/98 (8)	6/64 (9)	2/34 (6)	0.548
Altered neurocognitive tests**, n (%)	25/53 (47)	18/40 (45)	7/13 (54)	0.579
T-cell Activation				
CD38+CD8+%, median (IQR)*	13 (7–23)	12 (6–21)	17 (8–26)	0.074
CD45R0+CD38+CD8+%, median (IQR)*	8 (4–16)	7 (4–14)	11 (6–18(	0.017
T-cell Maturation/Differentiation				
CD127+CD4+%, median (IQR)*	11 (6–15)	11 (7–16)	9 (5–13)	0.059
CD127+CD8+%, median (IQR)*	26 (21–34)	26 (21–33)	25 (21–35)	0.656
CD4SRA+CD4+%, median (IQR)*	7 (3–10)	7 (4–10)	6 (3–8)	0.250
CD45RA+CD8+%, median (IQR)*	17 (13–23)	20 (14–24)	16 (10–19)	0.007
CD45R0+CD8+%, median (IQR)*	20 (16–29)	20 (15–29)	25 (16–32)	0.163

H-CSF, patients with CSF HIV-RNA ≥10.000 cp/mL; L-CSF, patients with CSF HIV-RNA<10.000 cp/mL.

